# Reactions of Cre with Methylphosphonate DNA: Similarities and Contrasts with Flp and Vaccinia Topoisomerase

**DOI:** 10.1371/journal.pone.0007248

**Published:** 2009-09-30

**Authors:** Chien-Hui Ma, Aashiq H. Kachroo, Anna Macieszak, Tzu-Yang Chen, Piotr Guga, Makkuni Jayaram

**Affiliations:** 1 Section of Molecular Genetics and Microbiology, University of Texas at Austin, Austin, Texas, United States of America; 2 Department of Bio-organic Chemistry, Center for Molecular and Macromolecular studies, Polish Academy of Sciences, Lodz, Poland; University of Minnesota, United States of America

## Abstract

**Background:**

Reactions of vaccinia topoisomerase and the tyrosine site-specific recombinase Flp with methylphosphonate (MeP) substituted DNA substrates, have provided important insights into the electrostatic features of the strand cleavage and strand joining steps catalyzed by them. A conserved arginine residue in the catalytic pentad, Arg-223 in topoisomerase and Arg-308 in Flp, is not essential for stabilizing the MeP transition state. Topoisomerase or its R223A variant promotes cleavage of the MeP bond by the active site nucleophile Tyr-274, followed by the rapid hydrolysis of the MeP-tyrosyl intermediate. Flp(R308A), but not wild type Flp, mediates direct hydrolysis of the activated MeP bond. These findings are consistent with a potential role for phosphate electrostatics and active site electrostatics in protecting DNA relaxation and site-specific recombination, respectively, against abortive hydrolysis.

**Methodology/Principal Findings:**

We have examined the effects of DNA containing MeP substitution in the Flp related Cre recombination system. Neutralizing the negative charge at the scissile position does not render the tyrosyl intermediate formed by Cre susceptible to rapid hydrolysis. Furthermore, combining the active site R292A mutation in Cre (equivalent to the R223A and R308A mutations in topoisomerase and Flp, respectively) with MeP substitution does not lead to direct hydrolysis of the scissile MeP bond in DNA. Whereas Cre follows the topoisomerase paradigm during the strand cleavage step, it follows the Flp paradigm during the strand joining step.

**Conclusions/Significance:**

Collectively, the Cre, Flp and topoisomerase results highlight the contribution of conserved electrostatic complementarity between substrate and active site towards transition state stabilization during site-specific recombination and DNA relaxation. They have potential implications for how transesterification reactions in nucleic acids are protected against undesirable abortive side reactions. Such protective mechanisms are significant, given the very real threat of hydrolytic genome damage or disruption of RNA processing due to the cellular abundance and nucleophilicity of water.

## Introduction

**Figure 1:**
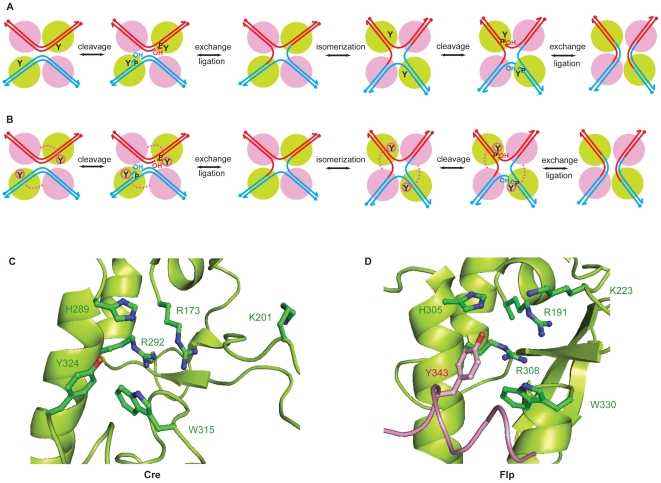
Reaction pathways and active site configurations for tyrosine site-specific recombination promoted by Cre and Flp. A, B. Recombination by Flp and Cre proceeds through two steps of single strand exchanges via a Holliday junction intermediate. During strand cleavage, the pair of recombinase monomers in green activates the scissile phosphodiester bonds in *cis* for nucleophilic attack by the catalytic tyrosine. The pair of Flp monomers in magenta donates the tyrosine nucleophile in *trans*. The corresponding Cre monomers are thought to allosterically confer chemical competence on the green monomers that perform strand cleavage in *cis*. B, C. Cross-sections of the Cre and Flp active sites containing the catalytic pentad and the tyrosine nucleophile are shown [5], [6]. In Flp, this tyrosine (shown in magenta) is donated by the neighboring Flp monomer.

Tyrosine site-specific recombinases, which include the Cre recombinase of bacteriophage P1 and the Flp recombinase of *Saccharomyces cerevisiae*, follow the type IB topoisomerase chemistry to promote DNA breakage and union [1]–[3]. During strand cleavage, the active site tyrosine nucleophile becomes covalently linked to the 3′-phosphate end of the broken DNA [3], [4]. The 5′-hydroxyl group generated by cleavage serves as the nucleophile for strand joining. Strand exchange follows from attack by the 5′-hydroxyl formed within a DNA substrate on the 3′-phosphotyrosyl bond formed within its partner. Recombination occurs within a synaptic structure containing four recombinase monomers and two DNA target sites. The reaction is completed in two steps of single strand exchanges via a Holliday junction intermediate (Figure 1A, B).

In addition to the tyrosine nucleophile, the Cre and Flp active sites are characterized by a catalytic pentad comprised of two arginines, a lysine, a histidine and a tryptophan [5], [6]. The type IB topoisomerases also display a similar catalytic constellation, except that the histidine and tryptophan positions are occupied by lysine and histidine, respectively [3], [7], [8]. There is a striking difference, though, between Cre and Flp in how they assemble their respective active sites. The Flp active site is shared between two neighboring recombinase monomers, one of which provides the pentad and the other the tyrosine nucleophile [5], [9]. As a result, strand cleavage by Flp occurs in a *trans* fashion, that is, across the strand exchange region from where the tyrosine donating Flp monomer is bound (Figure 1B, D). In Cre, the active site is housed entirely within a single monomer, and strand cleavage takes place in *cis* [3], [6]. A non-shared active site and cleavage in *cis* are also characteristic of the type IB topoisomerases. The strand cleavage and strand joining steps of recombination or DNA relaxation are thought to follow an acid/base assisted SN_2_ in-line nucleophilic substitution via a pentacoordinated transition state [10], [11]. The array of hydrogen bond donors surrounding the scissile phosphate helps stabilize the transition state, imparting a significant electrophilic character to the recombination and topoisomerase reactions [3].

Substitution of the negatively charged phosphate (P) by the charge-neutral methylphosphonate (MeP) group has been exploited successfully to investigate the importance of electrostatics in nucleic acid-protein interactions as well as catalytic mechanisms during DNA and RNA transactions [12]–[18]. Particularly relevant to this study are the effects of MeP modification at the scissile phosphate position, coupled with replacement of one of the two conserved arginine residues of the catalytic pentad, on the activities of vaccinia topoisomerase and Flp [19], [20]. Activation of the MeP bond is independent of Arg-223 of topoisomerase and the corresponding Arg-308 of Flp. The rate of strand cleavage by the tyrosine nucleophile (Tyr-274) in the MeP substrate is only two fold lower for Topo(R223A) compared to the wild type enzyme (see Table 1). The MeP-tyrosyl intermediate formed by cleavage, unlike its P-tyrosyl counterpart, is rapidly hydrolyzed by both wild type and mutant proteins. By contrast, MeP substitution, together with the R308A mutation, provokes a novel endonucleolytic activity in Flp that directly targets the scissile MeP linkage in DNA [19]. The reaction is independent of the Flp active site nucleophile Tyr-343. Direct hydrolysis of the scissile phosphodiester bond in DNA has not been observed with wild type Flp or Flp(R308A). To distinguish hydrolysis of the tyrosyl intermediate from that of the DNA phosphodiester, these two activities have been referred to as type I and type II endonucleases, respectively [19].

**Table 1:** First-order rate constants for strand cleavage in P- and MeP-half-sites and hydrolysis of the cleaved intermediate by Cre and Cre(R292A).

Recombinase/topoisomerase (Endonuclease Activity)	*k (s^−1^)*	P	MeP
Cre	*k_cl_*	1.2×10^−3^	–
	*k_hydrol_*	2.8×10^−5^	–
Cre(R292A)	*k_cl_*	–	3.2×10^−4^
	*k_hydrol_*	–	8.8×10^−5^
Flp	*k_cl_*	1.8×10^−1^	2.4×10^−4^
	*k_hydrol_*	6.4×10^−5^	1.3×10^−5^
Vaccinia topoisomerase	*k_cl_*	4.0×10^−1^	1.4×10^−3^
	*k_hydrol_*	2.2×10^−7^	7.0×10^−3^
Topo(R223A)	*k_cl_*	4.0×10^−6^	7.0×10^−4^
	*k_hydrol_*	–	3.5×10^−3^

The rate constants for strand cleavage (*k*_cl_) and hydrolysis of the tyrosyl intermediate (*k*_hydrol_) were estimated from data shown in Figures 3 and 4 and additional data from similar assays. The software package Prism (version 5.02) for Windows (GraphPad Software, Inc.) was used for obtaining the kinetic parameters. In the reaction scheme 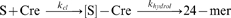
the first step of strand cleavage and formation of the Cre-DNA adduct is assumed to be irreversible. The trinucleotide product resulting from cleavage would diffuse away, and be unavailable for the back reaction. The values for the rate constants for Flp and vaccinia topoisomerase are taken from published work [19], [20].

The activities of Flp and topoisomerase, as well as their mutant derivatives, on P and MeP containing DNA substrates are suggestive of a potential role for phosphate or active site electrostatics in blockading water nucleophile during phosphoryl transfer in these two systems [19]–[21]. In the topoisomerase reaction, the strand joining step is inherently more vulnerable to potential hydrolysis than the strand cleavage step. Protection against this aberrant reaction is provided by the negative charge on the phosphate group, as inferred from the rapid hydrolysis of the MeP-tyrosyl intermediate by topoisomerase or Topo(R223A) under physiological pH. In the Flp reaction, it is the strand cleavage step that faces greater risk of hydrolysis than the strand joining step. Here, the positively charged Arg-308 appears to deter water, as indicated by the hydrolytic activity of Flp(R308A) on MeP-DNA.

The vaccinia topoisomerase and Flp paradigms may be relevant to several mechanistically similar phosphoryl transfer reactions in biological systems. They include DNA relaxation by type I and type II topoisomerases, conservative site-specific recombination, DNA transposition, telomere resolution in spirochetes, RNA splicing and retrohoming by mobile introns [10], [22]–[24]. The active nucleophiles in these reactions are a tyrosine or serine derived from the topoisomerase or recombinase or telomere resolvase active site or a 2′, 3′ or 5′ hydroxyl group derived from a nucleic acid or a nucleoside. It would seem logical that the evolutionary design of active sites performing these reactions, in the context of an overwhelming excess of water (∼56M), must have evolved strategies to avoid futile hydrolysis of the nucleic acid substrates or reaction intermediates.

As already noted, topoisomerase and Flp differ from each other in active site assembly (*cis* versus *trans*) and engagement of the scissile phosphate. Furthermore, the DNA relaxation complex and the recombination synapse are organized quite distinctly. The conformational dynamics associated with the two reactions are also discrete. These features may account for the characteristic responses of the two enzymes towards MeP containing DNA substrates. Cre is akin to topoisomerase, and contrary to Flp, in its mode of active site assembly and strand cleavage. However, Cre resembles Flp in the organization of its recombination synapse and the conformational dynamics of strand exchange [3], [4]. Two questions therefore become pertinent. Will Cre or Cre(R292A) unleash a strong hydrolytic activity, as topoisomerase and Topo(R223A) do, on the cleaved MeP-tyrosyl intermediate? Will Cre(R292A), like Flp(R308A), render the MeP bond in DNA susceptible to direct attack by water? Results presented in this report answer these questions. Hydrolysis of the MeP-tyrosyl bond by Cre or Cre(R292A) is not accelerated markedly compared to that of the P-tyrosyl bond by Cre. In this regard, Cre resembles Flp rather than topoisomerase. However, Cre(R292A) does not promote direct hydrolysis of the scissile MeP bond in DNA. In this respect, Cre is strikingly different from Flp, and quite like topoisomerase.

## Results

### General experimental designs for reactions of Cre on MeP substituted substrates

**Figure 2:**
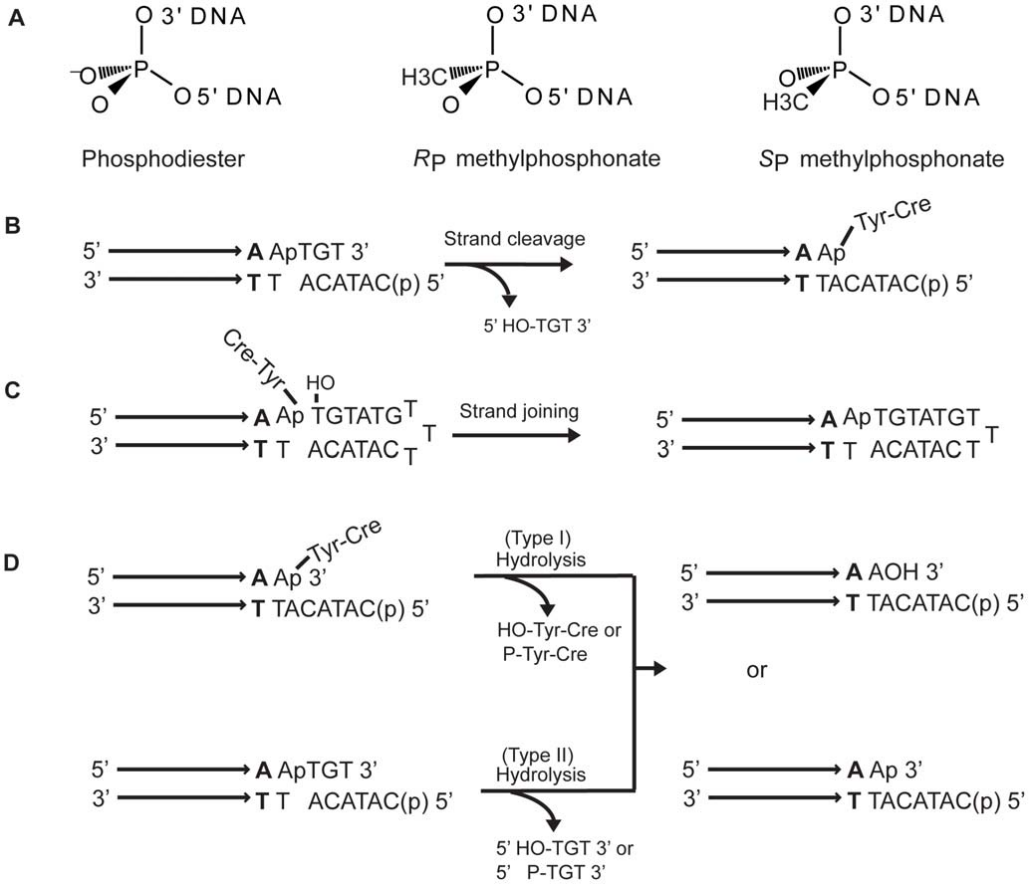
Half-site substrates for Cre reactions containing a phosphodiester or methylphosphonate at the scissile position. A. Substitution of a nonbridging oxygen atom of the achiral phosphodiester position in a DNA chain will generate either the *R*_P_ or *S*_P_ stereoisomer of methylphosphonate. B. In the schematic representation of a half-site substrate, the horizontal arrows represent a Cre binding element with its terminal bp abutting the spacer shown in bold. The scissile phosphodiester is indicated as ‘p’. Strand cleavage in a half-site traps the tyrosyl intermediate, as the trinucleotide product (5′HO-TGT3′) diffuses away. The 5′ end of the bottom strand is blocked by phosphorylation (denoted by ‘p’ in parentheses) from acting as the nucleophile in a joining reaction. C. A half-site designed to probe strand joining contains a longer bottom strand ending in a 5′-hydroxyl group. This hydroxyl could be positioned for nucleophilic attack in the cleaved intermediate by the single stranded region looping back as diagrammed. D. Possible products from hydrolysis of the tyrosyl intermediate resulting from strand cleavage (type I) or direct hydrolysis of the scissile phosphodiester in DNA (type II) are indicated. The presence of a hydroxyl or a phosphate group at the 3′ end in the hydrolytic product is determined by the line of attack of the water nucleophile.

MeP substituted DNA substrates are particularly instructive in assessing the role of phosphate and active site electrostatics in the mechanisms of type IB topoisomerase, Flp and related phosphoryl transfer reactions [19]–[21]. Substitution of either of the two non-bridging oxygen atoms of the scissile phosphate by the uncharged methyl group reduces the nominal negative charges in the ground and transition states from −1 to 0 and −2 to −1, respectively. The *R*_P_ and *S*_P_ stereoisomers of the chiral MeP with respect to the achiral P are diagrammed in Figure 2A. According to convention, replacement of the pro-*R* oxygen yields the *S*_P_ form MeP; that of the pro-*S* oxygen results in the *R*_P_ form MeP. The majority of reactions reported here were carried out with a mixture of the two MeP diastereomers; a subset of reactions was performed with stereochemically pure *R*_P_ or *S*_P_ form.

P- or MeP-’half-site’ substrates contain one Cre binding element and one cleavage site (Figure 2B, C). In the native full-site target of Cre, the spacer between the two Cre binding elements is 8 bp long, with the scissile phosphates on the two strands being 6 bp apart. Although a single monomer of Cre harbors a complete active site, crystal structures suggest that its functionality may require allosteric activation by a second monomer [6], [25]. Hence reactions are likely to occur in the context of at least two Cre bound half-sites. Cleavage of a half-site by Tyr-324 of Cre, with formation of the Cre-linked DNA intermediate (Figure 2B), was assayed by SDS-polyacrylamide gel electrophoresis (SDS-PAGE). Potential hydrolysis in a half-site, with either the DNA or the tyrosyl intermediate as target for water attack, was assayed by denaturing urea-polyacrylamide gel electrophoresis (denaturing PAGE). In most reactions, the 5′-OH on the bottom strand of the half-site was blocked by phosphorylation (Figure 2B) to prevent the strand joining reaction. A subset of the reactions utilized a half-site with a modified design in which this 5′ end was free (Figure 2C). In principle, the bottom strand could fold back to assist the hydroxyl group perform nucleophilic attack on the tyrosyl intermediate. This ‘pseudo strand exchange’ reaction within a half-site would produce a hairpin recombinant. Strand exchange is also possible between two half-sites to form a ‘pseudo-full-site’. The recombinant strands resulting from these reactions are identical in sequence, and will migrate as one band during electrophoresis under denaturing conditions. The potential Cre assisted hydrolytic reactions and the corresponding outcomes are indicated in Figure 2D. The target of hydrolysis could be the phosphotyrosyl intermediate (type I) or the scissile phosphodiester bond in DNA (type II). Previous studies have shown that both vaccinia topoisomerase and Flp exhibit the type I activity, whereas the type II activity is unique to Flp(R308A).

### Site-specific endonuclease activity of wild type Cre on native and MeP-half-sites

As pointed out, the presence of MeP at the scissile position is sufficient to turn vaccinia topoisomerase, but not Flp, into a strong endonuclease that targets the MeP-tyrosyl intermediate [19], [21]. Does MeP substitution induce Cre to behave like topoisomerase or like Flp?

**Figure 3:**
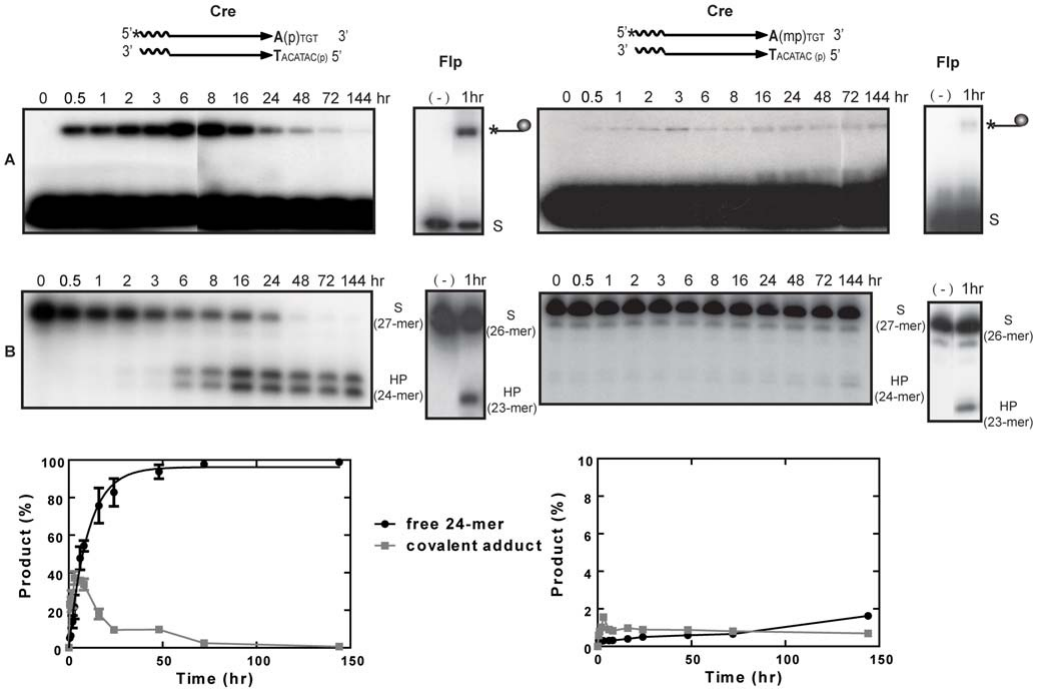
Strand cleavage and endonuclease activities of Cre in P- and MeP-half-site substrates. In the schematic representations of half-site substrates, the asterisk indicates ^32^P-label at the 5′-end, ‘p’ the scissile phosphate and ‘mp’ the scissile methylphosphonate. Reactions were split into halves to analyze the covalent Cre-DNA adduct (line ending in a circular knob) by SDS-PAGE (A) and the potential hydrolysis product(s) by denaturing PAGE (B). Reactions of Flp with its cognate half-sites in similar assays [19] are provided for comparison. The end-labeled half-site (A) or its labeled strand (B) is denoted by ‘S’ and the hydrolysis product by ‘HP’. The labeled strands in the Cre and Flp half-sites were 27 and 26 nucleotides long, respectively. HP in the Cre reaction was a 24-mer and that in the Flp reaction a 23-mer. The plots below represent mean values from three separate experiments with Cre.

The P- or MeP-half site (a racemic mixture of the *R*_P_ and *S*_P_ diastereomers) was treated with wild type Cre under standard conditions employed for recombination assays [26] at pH 7.5. Samples were split into halves, and were analyzed for the DNA-protein covalent adduct and the 24-mer hydrolysis product formed from the strand cleavage and potential endonuclease activities, respectively, of Cre (Figure 3A, B). Here and elsewhere, ‘strand cleavage’ refers exclusively to the reaction catalyzed by Tyr-324 of Cre to distinguish it from strand breakage mediated by water acting as an alternative nucleophile.

With the P-half-site, the covalent adduct formed early, peaking around 6–8 hr, and then steadily declined over the remainder of the reaction course (Figure 3A, left). Formation of the hydrolysis product (24-mer) trailed strand cleavage, increasing continuously and finally accounting for nearly quantitative conversion of the substrate (Figure 3A, right). Yields of the cleaved intermediate and the 24-mer from the MeP-half-site were much lower (Figure 3A and B, right) compared to the P half-site. However, the correlation between the kinetics of 24-mer formation and the decline of the tyrosyl intermediate was more or less similar between the two half-sites. To provide a frame of reference, the outcomes of reactions (1 hr time points) carried out with Flp on its cognate P- and MeP-half-sites are displayed alongside the corresponding Cre reactions.

The Cre reaction patterns suggest that the cleaved P-tyrosyl or MeP-tyrosyl intermediate is the precursor of the corresponding 24-mer hydrolysis product. This precursor-product relationship was also evinced by assays carried out with a P-full-site substrate (Figure S1, Supplemental Material). Consistent with this notion, neither the tyrosyl intermediate nor the 24-mer was formed in half-site reactions performed using the Cre(Y324G) mutant protein (Figure S2).

**Figure 4:**
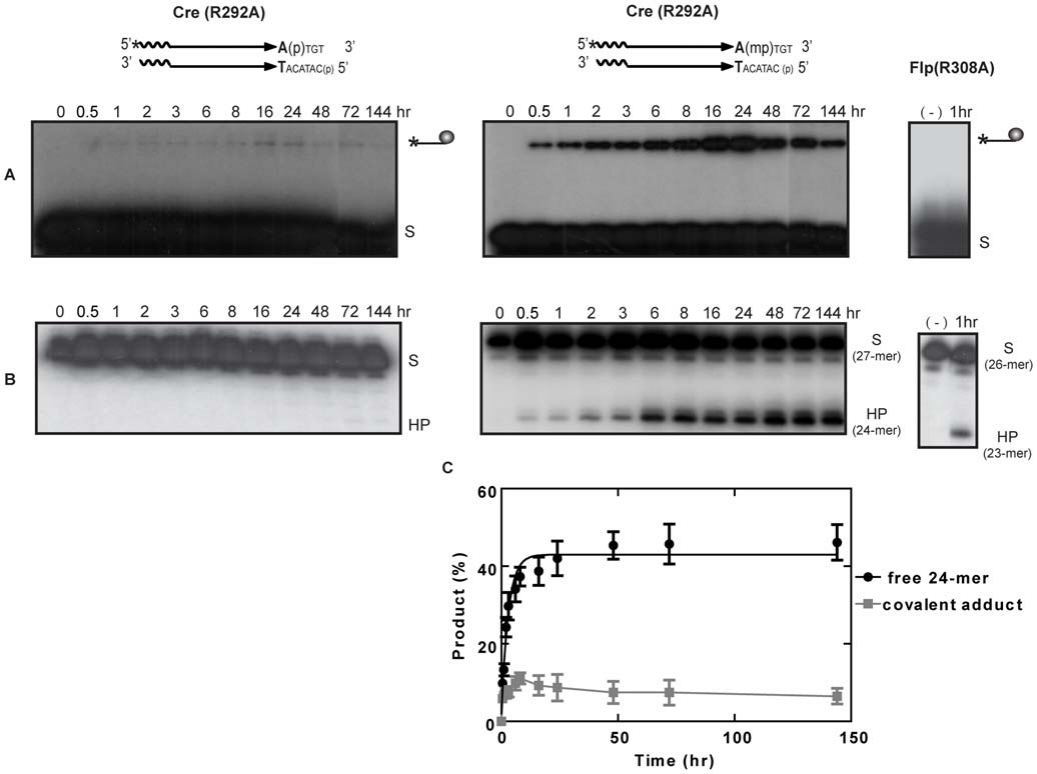
Reactions of Cre(R292A) assayed on P- and MeP-half-sites. Reactions and analysis of strand cleavage (A) and hydrolysis (B) products were performed as described under Figure 3. Reactions of Flp(R308A) [19] on the cognate MeP-half-site are shown for comparison.

The 24-mer product from the P-half-site reaction migrated as a roughly equal intensity doublet band (Figure 3B, left). As shown by further characterization (see below), the heterogeneity of the 24-mer was due to distinct 3′ ends, either a free hydroxyl group or a phosphate group. The hydrolysis product from the MeP-half-site was primarily a single band, corresponding to a 3′ methylphosphate end, although a fainter upper band was detectable at very late time points after prolonged phosphorimaging (Figure 3B, right). The 24-mer formation from the MeP-half-site was more abundant in reactions with a Cre mutant lacking the active site Arg-292 (see below; Figure 4).

The results summarized above demonstrate that the tyrosyl intermediate formed during Cre recombination is potentially subject to attack by water. However, hydrolysis does not become significant until several hours into the reaction. Under the conditions employed here, recombination between two P-full-sites goes to near completion in less than 30 min (data not shown). In a P-half-site containing a free 5′-hydroxyl group on its bottom strand, the predominant outcome was strand joining, not hydrolysis. The latter was not noticeable until about 3 hr into the reaction (Figure S3). There must be a built-in protective mechanism, other than phosphate electrostatics, to ensure that the 5′-hydroxyl group has a strong advantage over water as a nucleophile during strand joining (see under ‘Discussion’).

Cre is similar to Flp, and distinct from vaccinia topoisomerase, in that MeP substitution does not strongly enhance its endonucleolytic activity directed against the tyrosyl intermediate [19], [21] (Table 1). The efficiency of strand cleavage by Cre is significantly reduced in the MeP-half-site compared to the P-half-site. Once formed, the MeP-tyrosyl adduct does not turn over rapidly, but is hydrolyzed over hours (Figure 3B; [19]). By contrast, the rate of hydrolysis of the MeP-tyrosyl intermediate formed by topoisomerase is rapid compared to its P-tyrosyl counterpart [21] (Table 1). The half-life of the former at physiological pH is less than 2 minutes; that of the latter is roughly 36 days.

### Replacement of Arg-292 of Cre by alanine enhances strand cleavage in the MeP-half-site and correspondingly hydrolysis of the MeP-tyrosyl intermediate

Neutralizing the charge on the scissile phosphate by its replacement with MeP alleviates the requirement of Arg-223 in vaccinia topoisomerase and the corresponding Arg-308 in Flp for activating the MeP target for nucleophilic attack [19], [20]. Topo(R223A) forms the MeP-tyrosyl intermediate, and hydrolyzes it with roughly the same efficiency as the wild type enzyme. The reductions in the rates of strand cleavage and hydrolysis due to this mutation are within a factor of two (see Table 1). Flp(R308A), by contrast, directly hydrolyzes the MeP bond in DNA, preempting Tyr-343 mediated strand cleavage. Is Cre(R292A), the counterpart of Topo(R223A) and Flp(308A), competent for MeP activation? If it is, does it promote nucleophilic attack by Tyr-324 as in the topoisomerase case or direct hydrolysis (type II endonucleolytic activity) as in the Flp case?

Cre(R292A) was virtually nonreactive towards the P-half-site. Only a trace of the P-tyrosyl intermediate was detected (Figure 4A, left), and the 24-mer formation was also correspondingly inefficient. These results are consistent with the essential role of Arg-292 in the phosphate activation step. The MeP-half-site readily gave rise to the MeP-tyrosyl intermediate as well as the 24-mer in the Cre(R292A) reaction (Figure 4A and B, right). The MeP-tyrosyl intermediate reached its maximal levels at approximately 16–24 hr, and thereafter showed a slow, steady decay. The 24-mer continued to accumulate throughout the entire reaction course. In the Flp(R308A) reaction on its cognate MeP-half-site, shown here for comparison, the hydrolysis product (23-mer in this case) was formed directly without mediation of the MeP-tyrosyl intermediate [19].

**Figure 5:**
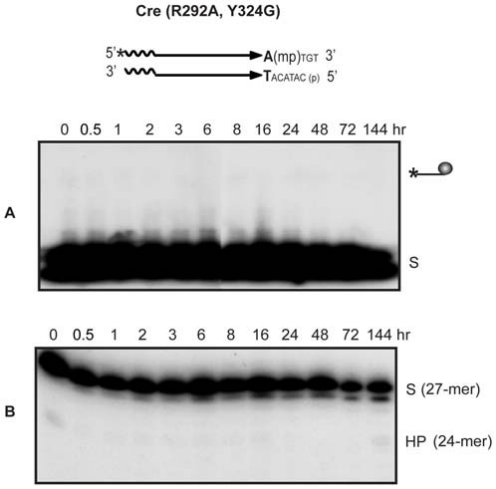
Lack of endonucleolytic activity in MeP-half-site reactions of Cre(R292A, Y324G). Reactions and product analysis were performed as described under Figure 3. The results of SDS-PAGE and denaturing PAGE analyses are shown in A and B, respectively. The predicted positions of migration of the covalent intermediate (line ending in a circular knob) and the hydrolysis product (HP) are indicated. There was a faint band of background hydrolysis in the control reaction, which did not increase in intensity with increasing time of incubation with Cre.

The kinetic data in Figure 4 are consistent with the 24-mer being the hydrolytic breakdown product of the MeP-tyrosyl intermediate. In accordance with their precursor-product relationship, treatment of the MeP-half-site with Cre(R292A,Y324G) did not result in the formation of either the cleaved intermediate or the 24-mer (Figure 5).

The endonucleolytic activity of Cre(R292A) on the scissile MeP bond is mechanistically similar to that of vaccinia topoisomerase, Topo(R223A) [20], [21] and the corresponding activity of wild type Flp, termed type I Flp endonuclease [19]. The Cre(R292A) activity is distinct from the type II endonuclease activity of Flp(R308A) whose target is the scissile MeP bond in DNA rather than the cleaved MeP-tyrosyl intermediate [19].

### Kinetic parameters for strand cleavage and hydrolysis of the cleaved intermediate by Cre, Flp and topoisomerase in P- and MeP-half-sites

Under our assay conditions, the first order rate constant for strand cleavage in the P-half-site by Cre was slower by a factor of ∼100 compared to Flp (*k*_cl_ of 1.2×10^−3^ s^−1^ versus 1.8×10^−1^ s^−1^) (Table 1). Because of the very low yields of strand cleavage and hydrolysis products from the MeP-half-site in Cre reactions, the corresponding rate constants were not determined. However, values of *k*_cl_ for the MeP-half-site were comparable between Cre(R292A) and Flp (3.2×10^−4^ s^−1^ and 2.4×10^−4^ s^−1^, respectively) (Table 1). The rate constant for hydrolysis of the MeP-tyrosyl intermediate by Cre(R292A) was ∼7 times that of Flp (*k*_hydrol_ of 8.8×10^−5^ s^−1^ versus 1.3×10^−5^ s^−1^) (Table 1). Note, though, that there was only about 3 fold enhancement in *k*_hydrol_ of the MeP-tyrosyl intermediate by Cre(R292A) relative to *k*_hydrol_ of the P-tyrosyl intermediate by Cre (8.8×10^−5^ s^−1^ and 2.8×10^−5^ s^−1^, respectively) (Table 1). The corresponding relative *k*_hydrol_ enhancement by the vaccinia topoisomerase or Topo(R223A) was much more dramatic, 1.6 to 3.2×10^4^ (*k*_hydrol_ of P-tyrosyl intermediate by topoisomerase  = 2.2×10^−7^ s^−1^; *k*_hydrol_ of MeP-tyrosyl intermediate by topoisomerase and Topo(R223A)  = 7.0×10^−3^ s^−1^ and 3.5×10^−3^ s^−1^, respectively) (Table 1).

Taken together, the results from Figures 3 and 4 and the kinetic constants in Table 1 argue against a significant role for either phosphate or active site electrostatics in guarding the strand cleaved intermediate formed by Cre against hydrolysis. They highlight the effect of eliminating Arg-292, critical for activity on the scissile phosphodiester bond, in enhancing the strand cleavage activity of Cre on the scissile MeP bond.

It should be noted that the methylphosphonates are not intrinsically prone to hydrolysis at neutral pH. Oligonucleotides containing MeP linkages are stable in concentrated ammonium hydroxide at 36° for up to 16 hr [27]. Direct hydrolysis of the scissile MeP bond by Flp(R308A) is significant against the absence of this reaction with Topo(R223A) and Cre(R292A). Similarly, the striking rate enhancement in the hydrolysis of the MeP-tyrosyl bond by topoisomerase and Topo(R223A) sharply differs from the lack of such enhancement by Cre, Cre(R292A), Flp and Flp(R308A).

### Stereospecificity of the Cre(R292A) endonucleolytic activity: characterization of the 3′-terminus of the hydrolysis product from P- and MeP-half-sites

Cre crystal structures indicate potential hydrogen bonding interactions between the two arginine residues of the catalytic pentad and the nonbridging oxygen atoms of the scissile phosphate [3], Substitution of either one of these oxygen atoms by a methyl group is likely to affect MeP reactivity. Furthermore, the magnitude of this effect could be different in the two cases. We therefore tested the potential stereochemical specificity of the Cre(R292A) endonucleolytic reaction using pure *S*_P_ and *R*_P_ forms of the MeP-half-site substrate. The hydrolysis product of Cre reaction on the P-half-site migrates as a doublet during electrophoresis whereas that of Cre(R292A) reaction on the MeP-half-site is almost exclusively a single band (Figures 3A and 4B). We wished to characterize these products with respect to their 3′ ends.

**Figure 6:**
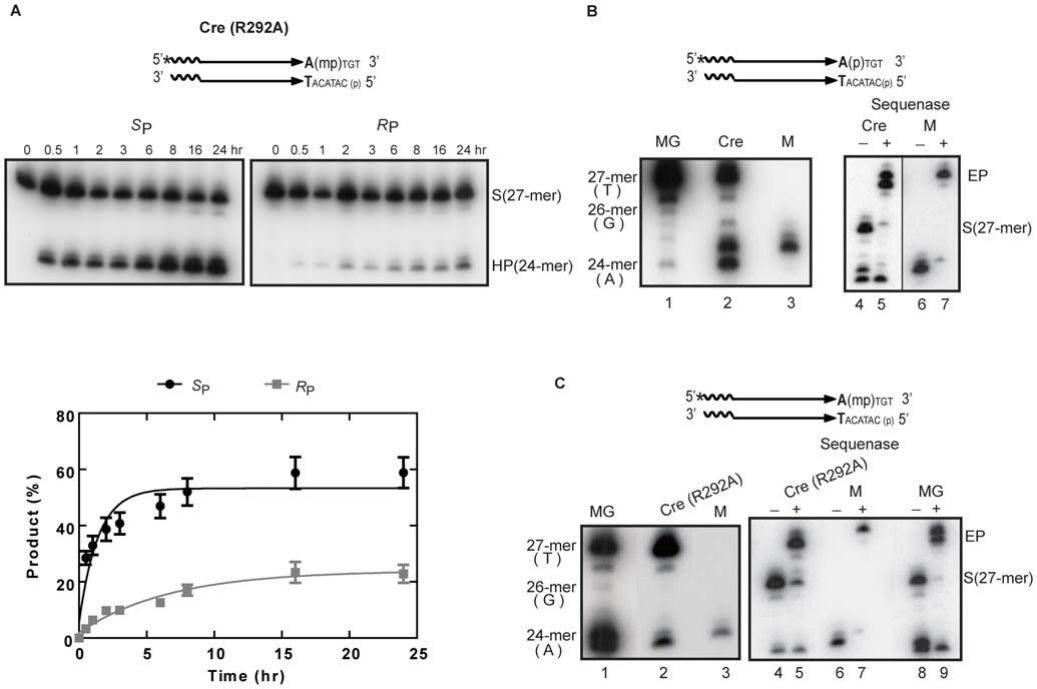
Stereochemical preference of Cre(R292A) in the MeP-half-site reaction and characterization of the hydrolysis products from P- and MeP-half-site substrates. A. The reactions of stereochemically pure *S*_P_ and *R*_P_ forms of the MeP-half-site with Cre(R292A) were analyzed by denaturing PAGE. B. The closely migrating hydrolysis products from the P-half-site (see Figure 3; lane 2 denotes a 24 hr reaction with Cre) were characterized for the chemical nature their 3′ ends. ‘MG’ refers to a Maxam-Gilbert sequencing ladder obtained by chemical modification at C+T positions followed by strand scission. The sizes of the oligonucleotides and the 3′-terminal bases they harbor are indicated against the rungs of the sequencing ladder. ‘M’ stands for a synthetic oligonucleotide marker (24-mer) with the expected sequence of the hydrolysis product and harboring a 3′-OH end. ‘EP’ refers to the extension product formed in a sequenase reaction. C. The hydrolysis product from a 6 hr Cre(R292A)/MeP-half-site reaction (lane 2) was subjected to similar analyses as in B. The marker oligonucleotide M was the same as that shown in B, harboring a hydroxyl group at the 3′ end. Strand breakage at the MeP position in the sequencing reaction produced a strong doublet (lanes 1 and 8). The top band of this doublet was extended in the elongation reaction, whereas the bottom band was not (compare lanes 8 and 9). In the sequenase reactions shown in B and C, the band marked S (27-mer) refers to the labeled top strands, carrying a 3′-hydroxyl end, of the P- or MeP-half-site. As expected, these oligonucleotides served as primers for sequenase.

Reactions of Cre(R292A) revealed a strong preference for the *S*_P_ MeP-half-site over the *R*_P_ form (Figure 6A). The *S*_P_ reaction showed an initial rapid phase of ∼30 min, followed by a slower phase. The *R*_P_ reaction followed a more uniform pace. For the early phase, the rate of product formation was roughly 7-fold higher for the *S*_P_ from. The product yield at the 24 hr time point favored the *S*_P_ over the *R*_P_ form by a factor of approximately 3. This bias in 24-mer formation was qualitatively the same as that reported for vaccinia topoisomerase and Flp. Since strand cleavage by the Tyr-343 nucleophile is accompanied by inversion of configuration at the phosphate center, the hydrolysis reaction targets the MeP-tyrosyl intermediate whose chirality is opposite to that of the starting *S*_P_ MeP-half-site.

The upper 24-mer band from Cre endonuclease activity (lane 2, Figure 6B) on the P-half site was determined to contain a hydroxyl group at the 3′ end by the following criteria. It migrated slightly above the appropriate rung of a Maxam-Gilbert sequencing ladder derived from the labeled strand of the half-site (lane 1 (MG), Figure 6B), and co-migrated with a synthetic 24-mer of the predicted sequence bearing a 3′-OH (lane 3 (M), Figure 6B). Furthermore, this hydrolysis product and the synthetic 24-mer served as primers in a sequenase primer extension reaction (lanes 5, 7, Figure 6B). By analogous reasoning, the lower 24-mer band was deemed to contain a 3′-phosphate end. It co-migrated with the appropriate band of the sequencing ladder and slightly below the synthetic 24-mer (lanes 1–3, Figure 6B), and failed to serve as a primer in the chain extension reaction (lane 5, Figure 6B). The relative mobility of the principal 24-mer band from Cre(R292A) reaction on the MeP-half-site (lane 2, Figure 6C) with respect to the sequence ladder (lane 1 (MG), Figure 6C) and the synthetic 24-mer marker (lane 3 (M), Figure 6C), as well as its failure to be extended by sequenase (lane 5, Figure 6C) in contrast to M (lane 7, Figure 6C), was consistent with its harboring a 3′-methylphosphate end. Note that chemical cleavage at the MeP position during the sequencing reaction yielded a doublet (lane 1, Figure 6C). The lower band was inferred to contain a 3′-methylphosphate end and the upper one a 3′-OH end, based on their behavior in the sequenase reaction (lane 9, Figure 6C).

Cre, Flp and vaccinia topoisomerase are mechanistically equivalent in that their hydrolytic actions on the respective MeP-tyrosyl intermediates generate products with a 3′-methylphosphate end, as the nearly exclusive or at least the major output. In these reactions, the water nucleophile is oriented opposite a tyrosine leaving group. Hydrolysis of the phosphotyrosyl intermediate by Flp and Cre yields products with either a 3′-OH or a 3′-phosphate end. It is possible that the hydroxyl end is generated by dephosphorylation. However, incubation of the isolated 24-mer with Cre for up to 8 hr did not remove the phosphate group from the 3′ end (Figure S4). By contrast, treatment with T4 polynucleotide kinase in the absence of ATP quantitatively dephosphorylated the 3′ end in 30 min. An intriguing alternative to dephosphorylation of the 24-mer during Cre reaction is for the attacking water molecule to line up opposite the 3′-OH leaving group. This would require considerable conformational flexibility within the active site. Both Flp and Cre have been shown to utilize a vicinal 2′-OH to attack the phosphotyrosyl bond when the scissile position is substituted with a ribonucleotide [28], [29].

## Discussion

We have examined the activities of the Cre recombinase and its active site variant Cre(R292A) on DNA half-site substrates containing MeP replacement of the scissile phosphate. We shall discuss the current results and their implications in the context of similar analyses carried out previously on vaccinia topoisomerase and Flp recombinase [19]–[21].

### The requirement of a conserved catalytic arginine in Cre, Flp and vaccinia topoisomerase is mandated by phosphate electrostatics

Arg-292 of Cre, like the corresponding conserved Arg-308 of Flp and Arg-223 of topoisomerase, becomes dispensable in activating the scissile phosphodiester bond when the negatively charged oxygen is replaced by the uncharged methyl group. In fact, Arg-292 poses serious impediment to MeP activation by Cre, as evinced by the ability of the R292A mutation to strongly stimulate strand cleavage in the MeP-half-site. Arg-223 of topoisomerase is more neutral with respect to MeP activation. The R223A mutation does not appreciably alter the rate of formation of the MeP-tyrosyl intermediate or of its subsequent hydrolytic breakdown [20], [21]. Unlike Cre and topoisomerase, Flp does not yield strand cleavage in the MeP substrate by Tyr-343 nucleophile when it harbors the R308A mutation. This is not due to lack of MeP activation; Flp(R308A) targets the MeP bond for direct hydrolysis (type II endonucleolytic activity) [19].

### Effect of MeP substitution on recombinase and topoisomerase activities: more than just active site perturbation?

The rates of strand cleavage by wild type topoisomerase and Flp in their MeP-substrates are reduced roughly by factors of 3×10^2^ and 10^3^, respectively, relative to the native P-substrates. [19], [20](see Table 1). The magnitude of this effect for Cre is likely to be in this range or perhaps even greater. Because of weak activity, we have not determined the rate constant for MeP-half-site cleavage by Cre.

The lower reactivity of the wild type recombinases and topoisomerase towards MeP may, at least in part, be accounted for by active site perturbations induced by the methyl modification. The larger van der Waals radius of the methyl group (∼1.8 Å) compared to the oxygen atom (∼1.5 Å), together with its non-polar character, could lead to steric clashes with the guanidinium group of Arg-292. Repositioning of the scissile MeP and/or active site residues to accommodate this conflict may thus result in disruption or weakening of contacts required for normal catalytic power. The stimulation of strand cleavage by the R292A mutation in Cre fits this explanation.

Nevertheless, removal of the arginine side chain may do more to catalysis than merely rectify the steric problem posed by MeP. For topoisomerase and Flp, the salutary effects of R223A and R308A mutations, respectively, on catalytic efficiency *per se* are not dramatic. The rate constants for strand cleavage in the MeP-substrate are within a factor of two for wild type topoisomerase (1.4×10^−3^ s^−1^) and Topo(R223A) (7×10^−4^ s^−1^) [20] (Figure 1). Strikingly, both proteins target the MeP-tyrosyl intermediate for rapid hydrolysis. Flp cleaves the MeP bond using the Tyr-343 nucleophile (2. 4×10^−4^ s^−1^) and Flp(R308A) hydrolyzes it directly (8.7×10^−5^s^−1^) with comparable rate constants [19] (Table 1). Thus methyl substitution by itself (as revealed by topoisomerase) or this substitution plus the removal of a catalytic arginine residue (as revealed by Flp(R308A) can dramatically alter the native reactions qualitatively.

### Protection against hydrolysis by phosphate and active site electrostatics?

Transesterification events involving phosphodiester bonds in DNA and RNA have to deal with the potential problem of water acting as an intrusive alternative nucleophile. The abortive hydrolysis of the scissile MeP bond by Flp(R308A) and that of the MeP-tyrosyl intermediate by vaccinia topoisomerase highlight the threat from water during site-specific recombination and DNA relaxation. These aberrant reactions induced by charge neutralization suggest that phosphate electrostatics or active site electrostatics may help repel water during the normal reaction. MeP substitution, in conjunction with specific active site mutations, could reveal related protective mechanisms in other enzyme systems that carry out phosphoryl transfer reactions in nucleic acids. Such mechanisms are expected to be modulated among individual enzyme systems according to their active site configurations as well as DNA-protein and protein-protein interactions and conformational dynamics within their reaction complexes. These aspects are considered below for Cre, Flp and topoisomerase.

### Guarding the scissile phosphodiester bond against hydrolysis during strand cleavage

**Figure 7:**
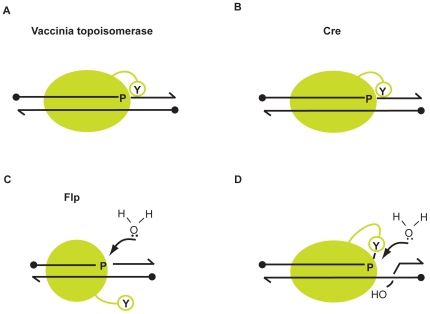
Mechanisms for the protection of the transesterification steps of DNA relaxation and site-specific recombination from hydrolysis. A, B. The strand cleavage steps of DNA relaxation by topoisomerase and recombination by Cre are protected by the temporal coupling between protein-DNA association and engagement of the scissile phosphate by the *cis*-acting active site. C. Binding of a Flp monomer to DNA activates the scissile phosphate but full engagement of the active site must await the binding of a second Flp monomer and donation of the active site tyrosine. Hydrolysis of the phosphodiester bond becomes a potential risk, which is minimized through active site electrostatics. D. During topoisomerase action, the cleaved tyrosyl intermediate is a potential target for attack by water during the strand rotation step that precedes ligation. The danger of hydrolysis at this stage is avoided through phosphate electrostatics.

Topoisomerase binds to DNA and relaxes it as a monomer. Flp and Cre also bind each of the two binding elements on their target sites as monomers. Because of their *cis* configuration, the topoisomerase and Cre active sites engage the scissile phosphodiesters contemporaneous to DNA binding. This would leave little chance for water to channel the cleavage reaction towards hydrolysis (Figure 7A and B). Whereas the topoisomerase monomer is fully competent for strand cleavage, the Cre monomer likely requires allosteric activation by a neighboring monomer to become cleavage proficient [3], [6].

Binding of a Flp monomer to DNA concomitantly activates the scissile phosphate [30]. However, it must await the binding of a second Flp monomer to acquire Tyr-343 and complete the assembly of its *trans* active site. The temporal separation between binding and active site engagement makes the scissile phosphate vulnerable to attack by water (Figure 7C). This risk appears to be minimized through the positively charged side chain of Arg-308. In Cre and topoisomerase, the role of Arg-292 and Arg-223, respectively, is purely catalytic; namely, activation of the scissile phosphate; in Flp, Arg-308 appears to play an additional non-catalytic role, namely, repelling or misorienting water from the activated phosphate.

Cre follows the topoisomerase, rather than Flp, paradigm to protect the strand cleavage reaction against hydrolysis.

### Protecting the tyrosyl intermediate against attack by water during strand joining

In the case of Cre, as is true for Flp as well, the susceptibility of the strand cleavage intermediate to hydrolysis is not particularly exacerbated by the presence of MeP at the scissile DNA position. This contrasts with the vaccinia topoisomerase reaction, in which the rate of hydrolysis of the MeP-tyrosyl intermediate is accelerated by a factor of ∼3 to 4×10^4^ relative to the p-tyrosyl intermediate [20], [21] (Table 1). During DNA relaxation, the cleaved strand must go through a round of rotation before joining occurs. The conformational flexibility associated with the dynamics of rotation may leave the phosphotyrosyl intermediate an open target for attack by water (Figure 7D). However, the negative charge on the phosphate keeps water out of the reaction center [20], [21].

Protection of the joining reaction from water during Cre and Flp recombination appears to be conferred by the compact design of their synaptic structures (see Figure 1A, B). The interface between two Flp monomers that partake in the *trans* donation of tyrosine and assembly of a functional active site displays extensive protein-protein interactions that bury a large surface area of ∼1500 Å^2^ [31]. The buried surface area in the Cre tetramer (∼1,100 Å^2^), though not as extensive, is nevertheless quite large [3], [6], [31]. Consistent with this difference perhaps, the hydrolysis product from full-site reactions appears earlier, and is more prominent, with Cre compared to Flp (this study; [19]). Crystal structures suggest that, within the tight synaptic architecture, strand cleavage and reorientation of the resulting 5′-hydroxyl groups in the strand joining orientation are strongly coupled events. The conformational dynamics associated with strand cleavage can effectively close the door to water.

Cre utilizes the Flp, rather than topoisomerase, strategy to protect the integrity of the strand joining reaction.

## Materials and Methods

### Synthetic oligonucleotides and assembly of substrates

A list of the oligonucleotides used for the assembly of half-site and full-site substrates is given in Table S1 (Supplementary data). All standard oligonucleotides were purchased from IDT (Coralville, IA). The methylphosphonate containing oligonucleotide (a racemic mixture of the *R*_P_ and *S*_P_ forms) was supplied by Trilink Technologies (San Diego, CA). Hybridization of oligonucleotides was performed as described previously [32].

### Stereochemically pure R_P_ and S_P_ methylphosphonate containing oligonucleotides

The oligonucleotide substrates containing *R*_P_ or *S*_P_ form of methylphosphonate were obtained as described previously [19]. Details of the individual steps of stereoisomer separation of MeP containing dinucleotides and oligonucleotide synthesis can be provided upon request.

### Purification of Cre and Cre mutants

Wild type Cre and its mutant derivatives, harboring a His-6 tag at their N-termini, were obtained as described previously [33]. Proteins were expressed in *Escherichia coli* BL21(DE3)pLysS strain by induction with 1 mM IPTG. They were purified from cell extracts by affinity chromatography on Ni Sepharose 6 Fast Flow columns (GE Healthcare, Piscataway, NJ). After buffer exchange through Econo-Pac 10DG columns (Bio-Rad, Hercules, CA), they were stored in 100 µl aliquots (containing 20% glycerol) at −70°C. The final purity of the protein preparations ranged from 65–80%, as judged by SDS-PAGE analysis.

### In vitro Cre reactions

In vitro Cre reactions were carried out using 5′-end labeled substrates at 30°C under conditions described by Ghosh et al. [26]. Each 30 µl reaction mixture contained 0.2 pmole end-labeled DNA substrate in 50 mM Tris-HCl, pH 7.5, 50 mM NaCl, 10 mM MgCl_2_ and 1 mM DTT. After addition of approximately 6–8 monomers of Cre or Cre mutant per Cre binding element, reaction mixtures were incubated at 30°C for various times. Reactions were terminated by addition of SDS to a final concentration of 0.1%. For assaying the Cre-DNA covalent adduct, samples were analyzed directly by SDS-PAGE. For assaying Cre endonuclease activity, the proteinase K treatment that normally precedes DNA extraction was omitted. By doing so, following electrophoresis, the hydrolysis product could be visualized without interference from the strand cleavage product carrying short Cre peptides at the 3′ end.

### Characterization of hydrolysis products by the sequenase extension reaction

Cre reactions were stopped by addition of SDS (0.1%), and samples were switched to the sequenase reaction buffer by passage over QIAquick PCR purification columns (QIAGEN). Extension reactions were done for 5 min at 37°C in the presence of all four deoxynucleoside triphosphates (0.5 mM final concentration), and analyzed by denaturing PAGE. The marker oligonucleotide or DNA purified (by phenol-chloroform extraction and ethanol precipitation) from the Maxam-Gilbert sequencing reactions was hybridized to the bottom stand of the half-site prior to treatment with sequenase.

### Dephosphorylation by T4 polynucleotide kinase

The T4 poly nucleotide kinase (New England BioLabs) reaction was performed using 10 units of the enzyme without addition of ATP. The incubation was done in the T4 poly nucleotide kinase reaction buffer provided by the supplier (70 mM Tris-HCl, 10 mM MgCl_2_, 5 mM DTT, pH 7.6 at 25°C) for 30 min at 37°C.

### Curve fitting to the kinetic data

Curves were fit to kinetic data by non-linear regression according to ‘one-phase-association’ using the software GraphPad Prism (version 5.02). For reactions that displayed early rapid kinetics followed by later slower kinetics, separation of the individual kinetic components by biphasic curve fitting was not attempted. First order rate constants were derived from the fitted curves based on the assumption that strand cleavage in the half-site substrates was irreversible (see also legend to Table 1).

### Quantification of reaction products

Bands corresponding to substrate and products were quantified using a phosphorimager. For low efficiency reactions, Conversion of substrate to product was quantified as follows. Multiple serial dilutions of a reaction were fractionated alongside the undiluted reaction, and the substrate band was quantified from those that fell within the linear range of estimation. Appropriate correction factors were then applied in the calculation of product yields.

## Supporting Information

Figure S1 Strand cleavage and endonucleolytic activities of Cre on a full-site substrate. In a schematic representation of the full-site substrate, the 32P-label at the 5′-end of the top strand and the scissile phosphates are indicated by the asterisk and ‘P’, respectively. Reactions were split into two equal portions, and analyzed for the covalent Cre-DNA adduct (line ending a circular knob) and the hydrolysis product (HP; 24-mer). SDS-PAGE and denaturing PAGE profiles are shown in A and B, respectively. In B, the mid-section of the gel, bereft of radioactive bands, was trimmed out. The end-labeled full-site (A) or its labeled strand (B) is denoted by ‘S’. The labeled strand was 54 nucleotides long. The results from two independent experiments are plotted below with error bars.
The nearly complete hydrolysis of the covalent intermediate under the reaction conditions employed was rather surprising. Nevertheless, the recombination reaction carried out under similar conditions goes to completion well before hydrolysis gains ground (data not shown). Even in a half-site reaction, a 5′-hydroxyl group present on the bottom strand strongly competes out water as the nucleophile in the strand joining reaction (see Figure S3).
Assays using the full-site 5′ end-labeled on the bottom strand yielded qualitatively similar results except that the hydrolysis reaction was not as strong as with the top strand labeled substrate. The maximal yield of the 24-mer product was approximately 20-30% of the input substrate (data not shown). This difference is perhaps due to the asymmetry in the cleavage of the scissile phosphodiester bonds on the two strands by Cre [1]–[7]. The direction of this asymmetry, towards the top or the bottom strand, is apparently influenced by whether cleavage occurs prior to or following synapsis of the recombination sites [2]. The cleavage reaction in full-sites is readily reversible by reformation of the parental strands or by strand exchange in the recombinant mode to form the Holliday junction. At early time points in our reactions (before its diminution by hydrolysis), the amount of covalent Cre-DNA complex formed on the top strand was greater than that on the bottom strand (data not shown). The longer-lived this intermediate is, the more susceptible it may become to attack by water. 1. Ennifar E, Meyer JE, Buchholz F, Stewart AF, Suck D (2003) Crystal structure of a wild-type Cre recombinase-loxP synapse reveals a novel spacer conformation suggesting an alternative mechanism for DNA cleavage activation. Nucleic Acids Res 31: 5449–5460. 2. Ghosh K, Lau CK, Gupta K, Van Duyne GD (2005) Preferential synapsis of loxP sites drives ordered strand exchange in Cre-loxP site-specific recombination. Nat Chem Biol 1: 275–282. 3. Hoess R, Wierzbicki A, Abremski K (1987) Isolation and characterization of intermediates in site-specific recombination. Proc Natl Acad Sci USA 84: 6840–6844. 4. Lee G, Saito I (1998) Role of nucleotide sequences of loxP spacer region in Cre-mediated recombination. Gene 216: 55–65. 5. Lee L, Sadowski PD (2003) Sequence of the loxP site determines the order of strand exchange by the Cre recombinase. J Mol Biol 326: 397–412. 6. Martin SS, Pulido E, Chu VC, Lechner TS, Baldwin EP (2002) The order of strand exchanges in Cre-LoxP recombination and its basis suggested by the crystal structure of a Cre-LoxP Holliday junction complex. J Mol Biol 319: 107–127. 7. Tribble G, Ahn YT, Lee J, Dandekar T, Jayaram M (2000) DNA recognition, strand selectivity, and cleavage mode during integrase family site-specific recombination. J Biol Chem 275: 22255–22267.
(1.87 MB TIF)

Figure S2 Reactions of Cre(Y324G) on phosphate (P) and methylphosphonate (MeP) half-site substrates. In the schematic diagram of the P- and MeP-half-sites, the scissile phosphodiester bonds are indicated by ‘p’ and ‘mp’, respectively. Reactions with Cre(Y324G) were analyzed as described under Figure S1. The expected positions of the Cre-DNA adduct (A; line ending in a circular knob) and the hydrolysis product (B; HP) are marked. S refers to the half-site (A) or its labeled 27-mer strand (B).
(2.18 MB TIF)

Figure S3 Competition between the 5′-OH and water nucleophiles during strand joining in a P-half-site. The half-site designed to follow strand joining contained a longer bottom strand than the standard half-sites shown in Figure S2. The 5′-hydroxyl group of this strand was left unblocked. Looping back of the single stranded region would place the 5′-hydroxyl in position to attack the tyrosyl intermediate formed by strand cleavage. This intra-half-site reaction would produce a hairpin (63-mer). Strand joining could potentially occur between two half-sites to yield a ‘pseudo-full-site’. The individual strands of the pseudo-full-site and the hairpin would be identical in sequence and display the same mobility during denaturing PAGE. ‘JP’ refers to this joined product, which was formed early and in greater abundance than the hydrolysis product (HP). The labeled strand of the half-site is denoted by ‘S’. The gel profile shown here was compressed by excluding its bare midsection.
(0.65 MB TIF)

Figure S4 Incubation of the 24-mer hydrolysis product with Cre does not cause 3′ end dephosphorylation. The 5′ end-labeled P-half-site was treated with Cre for 144 hr to convert it nearly quantitatively into the hydrolysis product via the cleaved intermediate. After phenol-chloroform extraction and ethanol precipitation, the isolated DNA was reincubated with Cre in the same buffer as that employed in the hydrolysis reaction. Incubation for up to 8 hr did not result in the removal of the 3′-phopshate (the lower 24-mer band). By contrast, incubation for 30 min with T4 polynucleotide kinase without addition of ATP led to complete removal of the phosphate group from the 3′ end.
(0.46 MB TIF)

Table S1 The synthetic oligonucleotides used for the assembly of the substrates used in this study are listed. *The bottom strand with a longer than normal 3′-extension was used to assemble the half-site substrate used in the strand joining assay (see Figure S3).
(0.03 MB DOC)
