# Supplementary figures and images for: Reactions of Cre with Methylphosphonate DNA: Similarities and Contrasts with Flp and Vaccinia Topoisomerase
Source: PLoS One. 2009 Sep 30;4(9):e7248. doi: 10.1371/journal.pone.0007248 (PMC2747268; doi:10.1371/journal.pone.0007248)

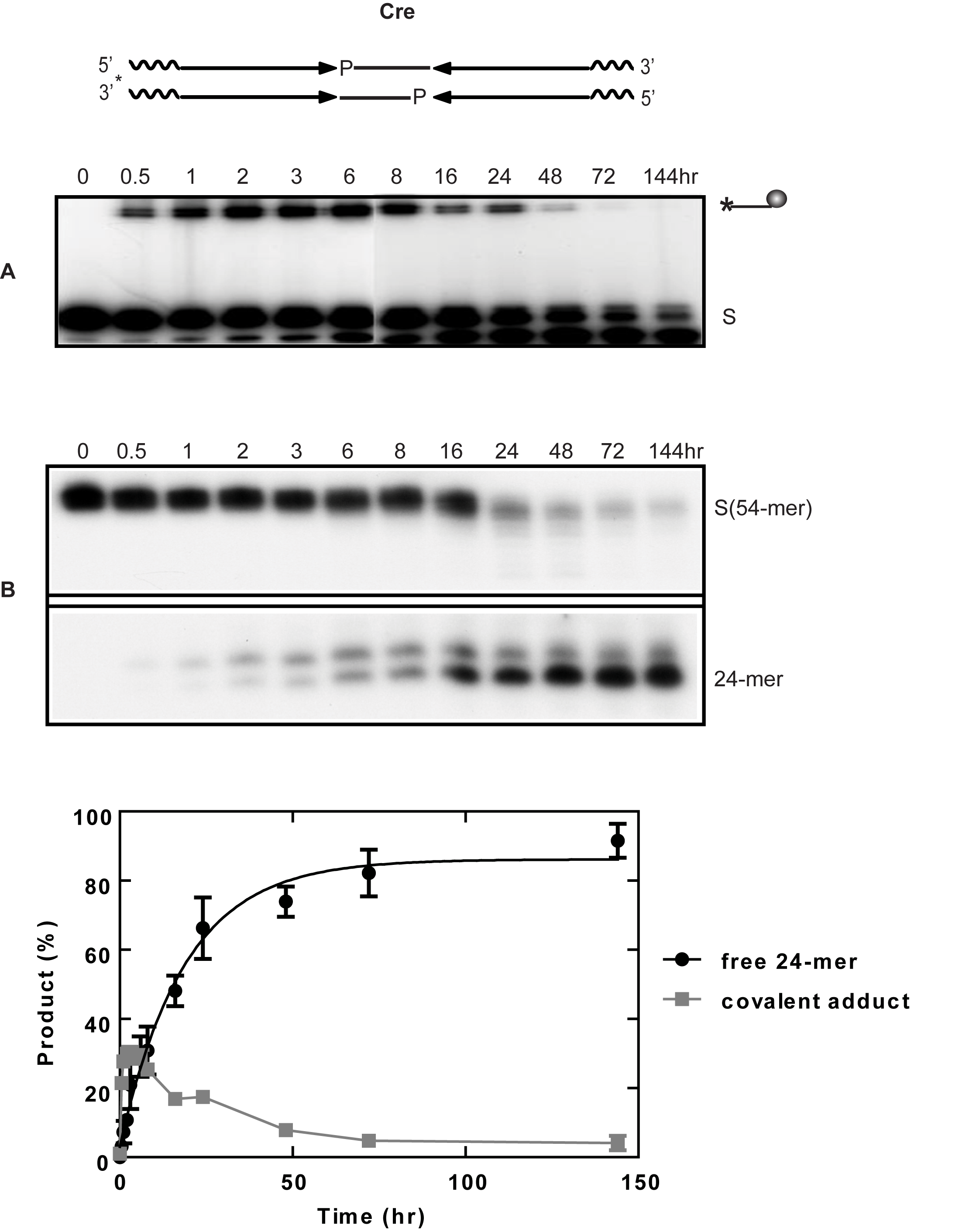

Supplement: Figure S1 — Strand cleavage and endonucleolytic activities of Cre on a full-site substrate. In a schematic representation of the full-site substrate, the 32P-label at the 5′-end of the top strand and the scissile phosphates are indicated by the asterisk and ‘P’, respectively. Reactions were split into two equal portions, and analyzed for the covalent Cre-DNA adduct (line ending a circular knob) and the hydrolysis product (HP; 24-mer). SDS-PAGE and denaturing PAGE profiles are shown in A and B, respectively. In B, the mid-section of the gel, bereft of radioactive bands, was trimmed out. The end-labeled full-site (A) or its labeled strand (B) is denoted by ‘S’. The labeled strand was 54 nucleotides long. The results from two independent experiments are plotted below with error bars. The nearly complete hydrolysis of the covalent intermediate under the reaction conditions employed was rather surprising. Nevertheless, the recombination reaction carried out under similar conditions goes to completion well before hydrolysis gains ground (data not shown). Even in a half-site reaction, a 5′-hydroxyl group present on the bottom strand strongly competes out water as the nucleophile in the strand joining reaction (see Figure S3). Assays using the full-site 5′ end-labeled on the bottom strand yielded qualitatively similar results except that the hydrolysis reaction was not as strong as with the top strand labeled substrate. The maximal yield of the 24-mer product was approximately 20-30% of the input substrate (data not shown). This difference is perhaps due to the asymmetry in the cleavage of the scissile phosphodiester bonds on the two strands by Cre [1]–[7]. The direction of this asymmetry, towards the top or the bottom strand, is apparently influenced by whether cleavage occurs prior to or following synapsis of the recombination sites [2]. The cleavage reaction in full-sites is readily reversible by reformation of the parental strands or by strand exchange in the recombinant mode to fo [file pone.0007248.s001.tif]

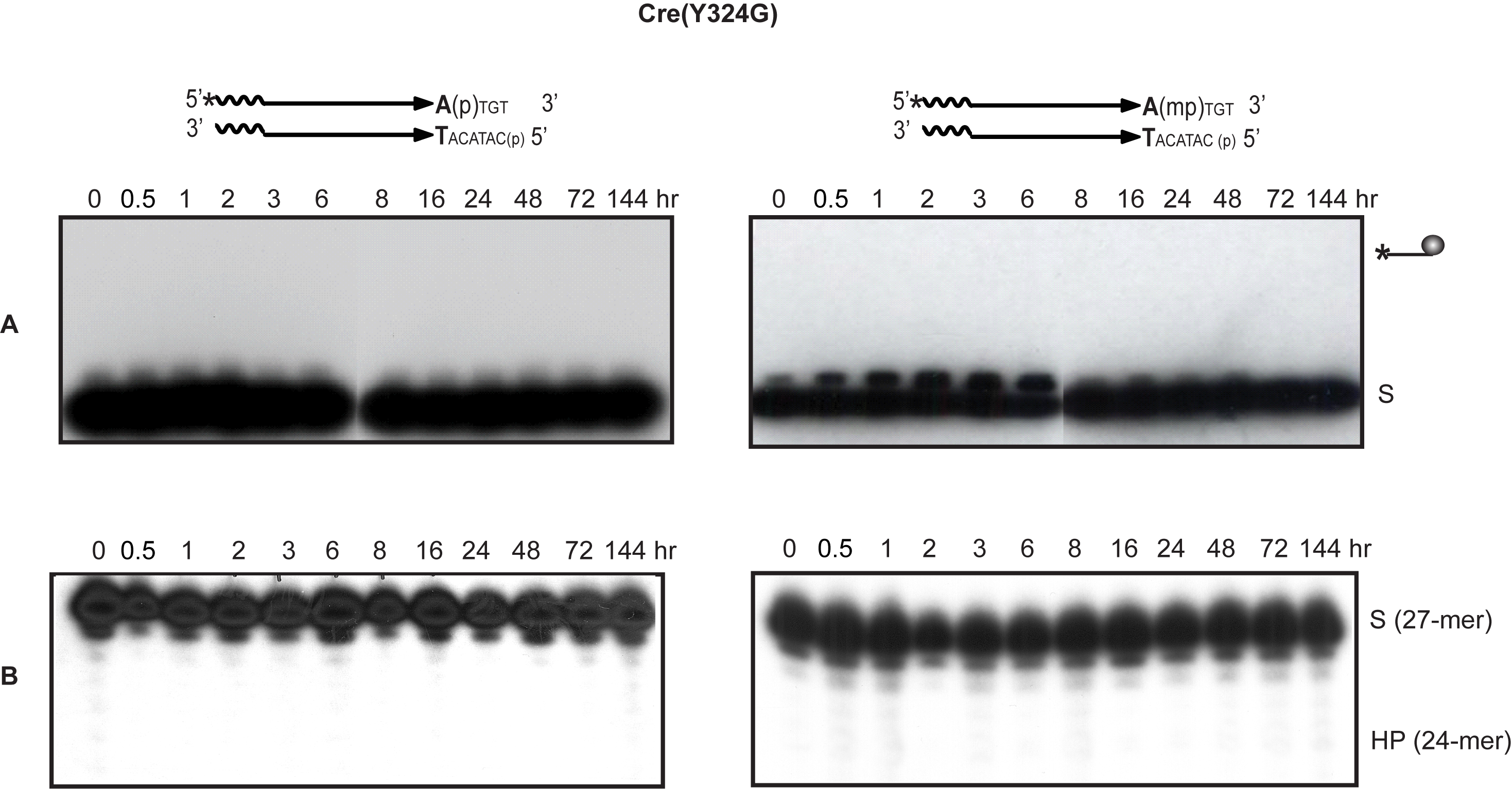

Supplement: Figure S2 — Reactions of Cre(Y324G) on phosphate (P) and methylphosphonate (MeP) half-site substrates. In the schematic diagram of the P- and MeP-half-sites, the scissile phosphodiester bonds are indicated by ‘p’ and ‘mp’, respectively. Reactions with Cre(Y324G) were analyzed as described under Figure S1. The expected positions of the Cre-DNA adduct (A; line ending in a circular knob) and the hydrolysis product (B; HP) are marked. S refers to the half-site (A) or its labeled 27-mer strand (B). (2.18 MB TIF) [file pone.0007248.s002.tif]

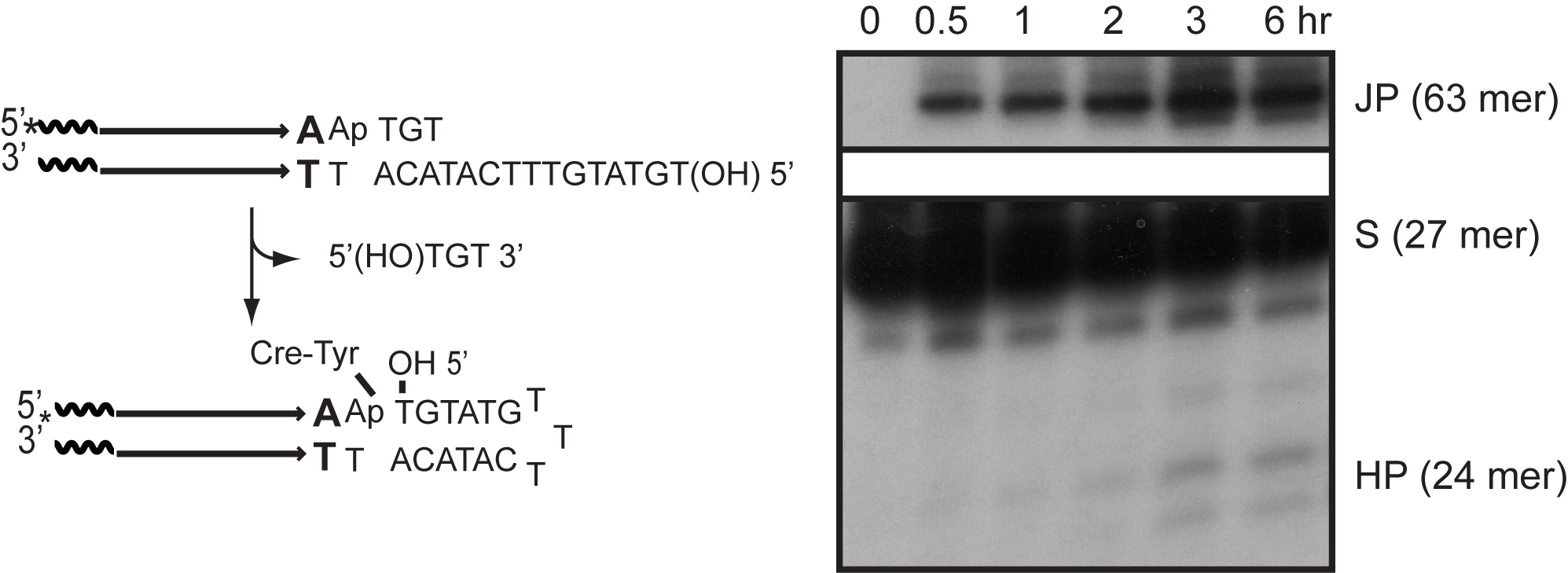

Supplement: Figure S3 — Competition between the 5′-OH and water nucleophiles during strand joining in a P-half-site. The half-site designed to follow strand joining contained a longer bottom strand than the standard half-sites shown in Figure S2. The 5′-hydroxyl group of this strand was left unblocked. Looping back of the single stranded region would place the 5′-hydroxyl in position to attack the tyrosyl intermediate formed by strand cleavage. This intra-half-site reaction would produce a hairpin (63-mer). Strand joining could potentially occur between two half-sites to yield a ‘pseudo-full-site’. The individual strands of the pseudo-full-site and the hairpin would be identical in sequence and display the same mobility during denaturing PAGE. ‘JP’ refers to this joined product, which was formed early and in greater abundance than the hydrolysis product (HP). The labeled strand of the half-site is denoted by ‘S’. The gel profile shown here was compressed by excluding its bare midsection. (0.65 MB TIF) [file pone.0007248.s003.tif]

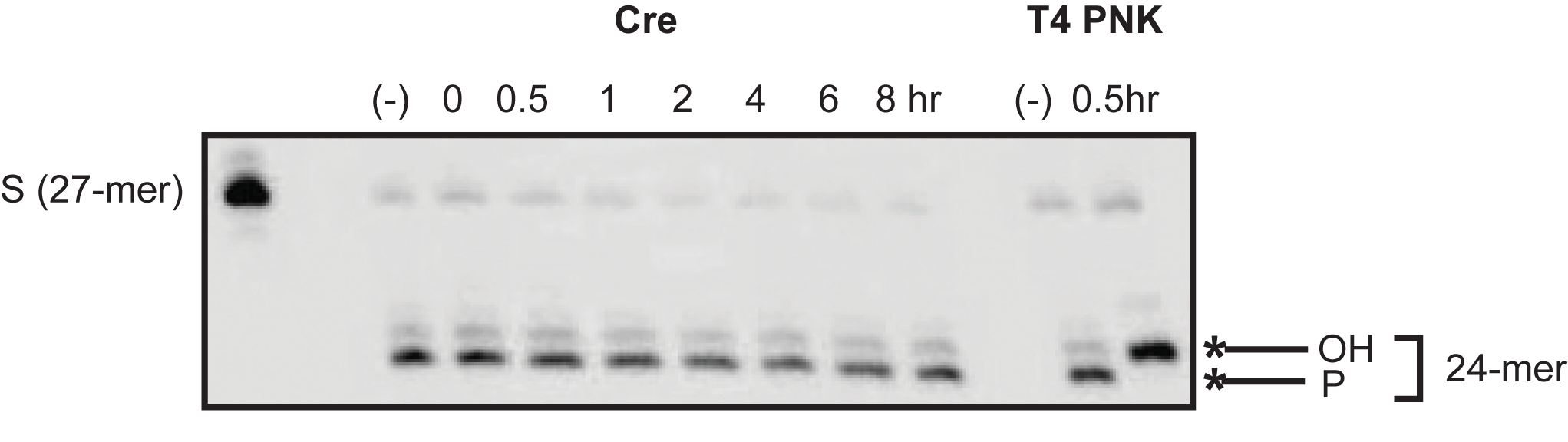

Supplement: Figure S4 — Incubation of the 24-mer hydrolysis product with Cre does not cause 3′ end dephosphorylation. The 5′ end-labeled P-half-site was treated with Cre for 144 hr to convert it nearly quantitatively into the hydrolysis product via the cleaved intermediate. After phenol-chloroform extraction and ethanol precipitation, the isolated DNA was reincubated with Cre in the same buffer as that employed in the hydrolysis reaction. Incubation for up to 8 hr did not result in the removal of the 3′-phopshate (the lower 24-mer band). By contrast, incubation for 30 min with T4 polynucleotide kinase without addition of ATP led to complete removal of the phosphate group from the 3′ end. (0.46 MB TIF) [file pone.0007248.s004.tif]
