# Supplementary material for: Reactions of Cre with Methylphosphonate DNA: Similarities and Contrasts with Flp and Vaccinia Topoisomerase
Source: PLoS One. 2009 Sep 30;4(9):e7248. doi: 10.1371/journal.pone.0007248 (PMC2747268; doi:10.1371/journal.pone.0007248)
Supplement: Table S1 — The synthetic oligonucleotides used for the assembly of the substrates used in this study are listed. *The bottom strand with a longer than normal 3′-extension was used to assemble the half-site substrate used in the strand joining assay (see Figure S3). (0.03 MB DOC) [file pone.0007248.s005.doc]

| **OLIGOMER** | **Sequence** |
| --- | --- |
| **P-half-site (top strand)** | 5' ACTTGGATCC**ATAACTTCGTATA**ATGT  3' |
| **MeP-half-site (top strand)** | 5' ACTTGGATCC**ATAACTTCGTATA**A (mp)TGT  3' |
| **P- or MeP-half-site (bottom strand)** | 5' CATacattatacgaagttatggatccaagt 3' |
| **P-half-site (bottom strand*)** | 5’ TGTATGTTTCATACATTATACGAAGTTATGGATCCAAGT 3’ |
| **MeP-half-site (*S*p top strand)** | 5' ACTTGGATCC**ATAACTTCGTATA**A (mp)TGT  3' |
| **MeP-half-site (*R*p top strand)** | 5' ACTTGGATCC**ATAACTTCGTATA**A (mp)TGT  3' |
| **MeP-half-site (*S*p or *R*p bottom strand)** | 5' acattatacgaagttatggatccaagt 3' |
| **P-full-site** | 5' ACTTGGATCC **ATAACTTCGTATA**atgtatgc**TATACGAAGTTAT**acttggatcc 3' |
| **P-full-site (bottom strand)** | 5’ GGATCCAAGTATAACTTCGTATAGCATACATTATACGAAGTTATGGATCCAAGT 3’ |
